# Room Temperature Crystallization of Hydroxyapatite in Porous Silicon Structures

**DOI:** 10.1186/s11671-016-1658-4

**Published:** 2016-11-10

**Authors:** M. Santana, J. O. Estevez, V. Agarwal, R. Herrera-Becerra

**Affiliations:** 1 Institute of Physics, UNAM, Circuito de la Investigación Científica Ciudad Universitaria, México, C. P. 04510 México; 2 Center for Engineering and Applied Sciences (CIICAp-UAEM), Av. Universidad 1001. Col. Chamilpa, Cuernavaca, Morelos 62209 México; 3 Posgrado en Ciencia e Ingeniería de Materiales, Circuito de la Investigación Científica, Ciudad Universitaria, Mexico, 04510 Mexico

**Keywords:** Porous silicon, Hydroxyapatite, Co-precipitation method

## Abstract

Porous silicon (PS) substrates, with different pore sizes and morphology, have been used to crystallize hydroxyapatite (HA) nano-fibers by an easy and economical procedure using a co-precipitation method at room temperature. In situ formation of HA nanoparticles, within the meso- and macroporous silicon structure, resulted in the formation of nanometer-sized hydroxyapatite crystals on/within the porous structure. The X-ray diffraction technique was used to determine the tetragonal structure of the crystals. Analysis/characterization demonstrates that under certain synthesis conditions, growth and crystallization of hydroxyapatite layer on/inside PS can be achieved at room temperature. Such composite structures expand the possibility of designing a new bio-composite material based on the hydroxyapatite and silicon synthesized at room temperature.

## Background

Porous silicon is a nanostructured material obtained by electrochemical anodization of monocrystalline silicon (c-Si) in a solution of hydrofluoric acid and ethanol [1]. One of the most important characteristics of PS is its high specific surface area [1–3], which was shown to have a nanostructured fractal (self-similar) surface [4] with tunable interconnected pores. It serves as an ideal substrate for crystal growth of different materials such as proteins [5, 6], oxides [7], semiconductors [8], metallic nanoparticles [9], and hydroxyapatites [2, 10]. Moreover, heterogeneous nucleation occurs more often than homogeneous nucleation. In different studies, the pore filling via nucleation inside the pore itself or nucleation from a branching pore has been demonstrated [11]. Such fractal surface features have a great impact on the properties of different nanostructured materials adsorbed or grown over it [1–3]. The high specific surface area of porous silicon makes it highly reactive as well as biocompatible and biodegradable [1, 2]. Such characteristics provide a variety of applications such as sensors based on both electrical and optical properties, or in various medical applications such as intelligent drug delivery in the body [1, 2], and bone implants [10].

In vitro studies, involving the immersion of various materials in simulated body fluids [12–15], have used suitable porosities of mesoporous silicon in the formation of physiologically stable hydroxyapatite on its surface [16, 17]. Additionally, it has been used as surface substrates for cell culture based on hydroxyapatite-PS [18] and intelligent implants [19]. For obtaining composites based on PS and HA, different deposition methods have been proposed [20–22]. In addition, PS can be easily integrated into conventional electronics making feasible the possibility of developing smart bio-devices based on hydroxyapatite [1–3, 23].

On the other hand, HA is the principal constituent of the bone and has been used to induce bone and teeth formation at particular biological sites requiring bone repair and crystalline growth on composite-metallic substrates [24–26]. HA is present in the mineralized tissues in the form of microscopic crystals of impure ultrastructural complexes (such as enamel), with crystal size of approximately 1 μm long and 50 nm in diameter. As an example, in dentin and bone tissues, the HA crystal size is smaller than enamel [27]. From the dental standpoint, the crystalline orientation in the enamel is interesting due to the dissolution of the crystals in the process of cavity formation [28]. Consequently, the properties of HA are directly influenced by particle size and morphology [29]. The influence of the preparation methods on the chemical properties of HA is crucial, mainly due to its stability in a wide range of compositions, as well as accepting a variety of anionic and cationic substitutions. As a consequence of this, its behavior as biomaterial in a composite form can be easily modified for its development in tissue engineering [30–34] and drug delivery [35–37]. As HA morphology is sensitive to the preparation conditions, obtaining HA particles with desired characteristics could be tailored by appropriate selection of the synthetic pathway and the type of substrates in which HA can grow.

In this paper, we review our experimental results on enhanced infiltration, adhesion, nucleation, and crystallization of biological and inorganic materials in meso- and macroporous silicon. The phenomena of enhanced nucleation and crystallization of biological and inorganic materials on porous silicon are explained in terms of the level of surface fractality as well as pore size and shape. The proposed method is a new way to obtain HA-PS composites at room temperature.

## Methods

### Mesoporous Silicon

Nanostructured PS was obtained by the electrochemical etching of n- and p-type, (100) oriented silicon, with a resistivity of 0.001–0.005 and 0.002–0.005 Ω·cm, respectively. To study the nucleation of HA within the n-type PS template, anodization was carried out under illumination (254 nm), with electrolyte consisting of a mixture of aqueous 48 wt.% HF (hydrofluoric acid) and absolute ethanol (99.9 %) in a volumetric ratio of 2:1, respectively. The PS layer was obtained by applying a constant current density of 80 mA/cm^2^ for 2 min. p-type boron-doped silicon was etched at a constant current density of 70 mA/cm^2^ for 2 min, with an electrolyte consisting of HF and ethanol in the volumetric ratio of 1:1. All the PS templates were thermally oxidized in air at 300 °C.

### Macroporous Silicon

Macroporous silicon substrates were obtained by electrochemical dissolution of low doped, 8–12 Ω·cm, (100) oriented, n-type single-side polished crystalline Si substrate. A mixture of 48 wt.% aqueous HF and absolute ethanol in volumetric ratio of 1:4 was used as electrolyte to perform the etching process for 10 min. Samples were fabricated under the influence of electric and magnetic fields applied simultaneously. Experimental configuration consisted of n-type Si substrates with ohmic contacts prepared by rubbing Ga-In eutectic at the two extreme ends of the silicon wafer (30 × 10 mm). A lateral current flow (*I*_*x*_ = 100 mA) was applied across the Si substrate, while a platinum electrode was joined to the negative terminal (cathode) of the applied lateral current (across the Si sample). A magnetic field *H*_*y*_ = 0.4 T was placed perpendicular to the direction of the current (*I*_*x*_), so that the majority charge carriers (electrons, e^−^) flowing in the *x*-direction will be swept down by the effect of the resulting Lorentz force, leading to a major accumulation of valance band holes (h^+^) at the HF-silicon interface, promoting the reaction. On the other hand, the lateral electric field contributes to the formation of a structural gradient across the sample in terms of pore size (from approximately 3 μm to 500 nm). The abovementioned macroporous silicon formation process has been described in detail by Antunez et al. [38].

### HA Synthesis

On the other hand, HA samples were synthesized by co-precipitation method, with the following reagents: Ca(NO_3_)_2_•4H_2_O (Sigma-Aldrich, 99 %), N(C_3_H_7_)4OH (Sigma-Aldrich, 25 %), distilled water, and H_3_PO_4_ (Sigma-Aldrich, 85 %), following the stoichiometry of the chemical reaction reported in references [29, 39]. In this work, the following solutions were prepared: tannic acid 2 %; calcium chloride dihydrate 0.2 M; phosphoric acid 0.12 M; tetrapropyl ammonium hydroxide 0.2 M, and calcium nitrate tetrahydrate 0.2 M. The phosphoric acid was adjusted to pH 9 with tetrapropyl ammonium hydroxide via a gradual process. Aqueous solutions were prepared, mixed with constant stirring of 300 rpm, and reduced. The HA was synthesized in aqueous solution and was subsequently dried by a freeze-drying technique to obtain a powder as a final product (denoted as HA powder sample).

**Fig. 1:**
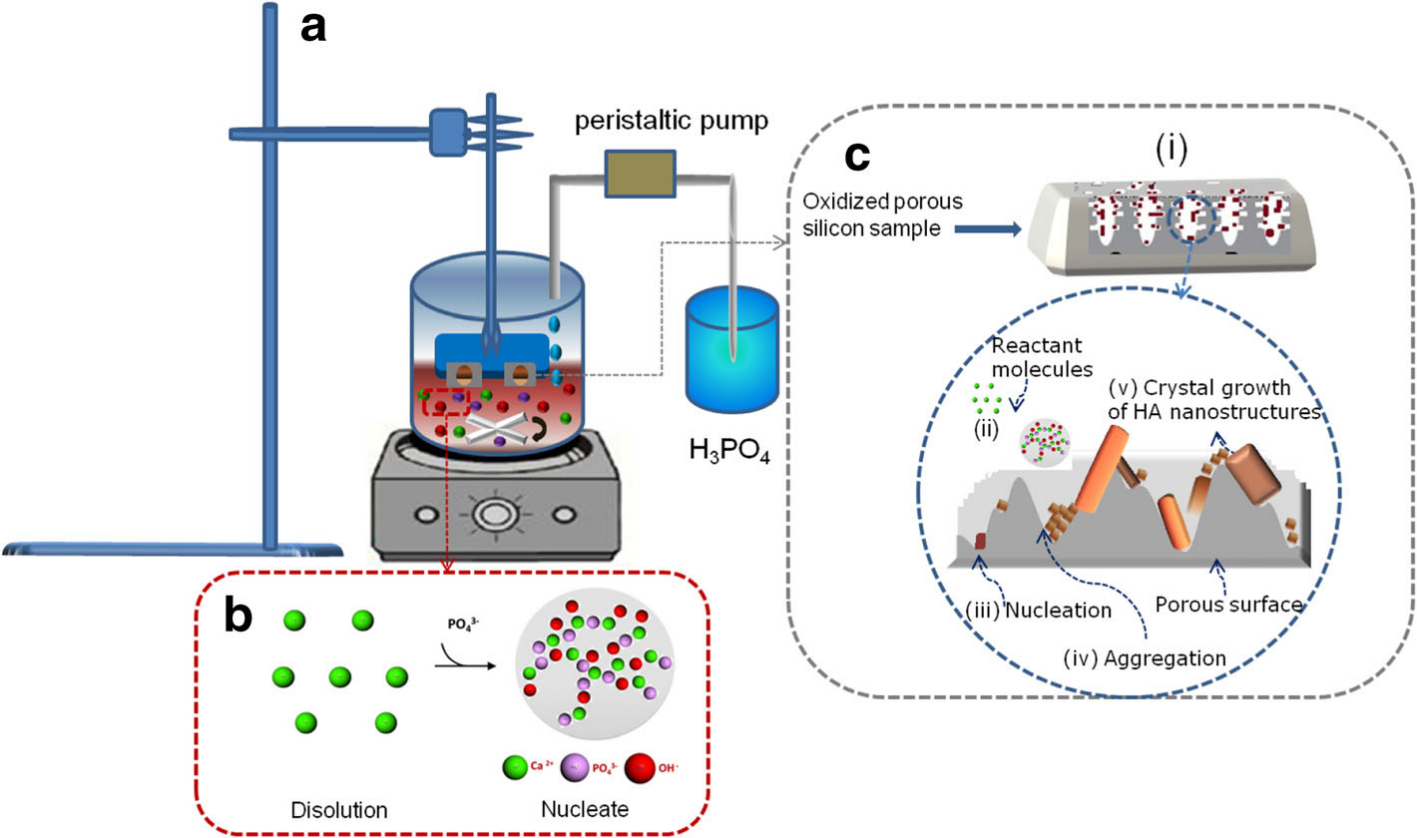
**a** Schematic illustration for growth of HA nanoparticles on PS by co-precipitation method. **b** Mixing of reactant for nucleation of HA. **c** Meso- and macroporous silicon substrate used to crystallize HA nanoparticles. Substrate roughness dependent formation of different morphologies of the crystals grown from the pore corners is explained in terms of the minimization of the surface free energy of the Si/SiO_2_ porous fractal surface as a result of the pore formation

For obtaining the composite HA@PS-n, HA@PS-p and MPS-HA, samples were immersed vertically in a solution containing the abovementioned precursors (schematically shown in Fig. 1a). The HA precursor solution kept in contact with the bottom half of the porous layer and infiltrates in the upper half by capillarity throughout the porous structure. Although the bottom part of the PS template is fractured due to the constant stirring for 24 h, the well-structured composite is formed within/over the upper half of the PS template through capillary action of the precursor solutions. Subsequently the sample obtained in aqueous solution is washed three times with a mixture of methanol-distilled water (1:2). The porous silicon-HA composite is washed with a dilute solution of methanol for further characterization.

### Characterization

The synthesized powders, HA@PS and MPS-HA samples were characterized by different techniques. X-ray diffraction (XRD) analysis was performed using a Bruker AXS D8 Advance diffractometer, with CuKα radiation (2*θ* from 4° to 110° with a step of 0.019°). Raman spectroscopy (Thermo Scientific) with confocal microscope and a 532-nm laser excitation source (10 mW) was used to measure the functional groups in the range of 3000–50 cm^−1^. For further analysis of structure and crystallinity, transmission electron microscopy (TEM) of HA powders was performed through a JEOL JEM-2010F FasTem. Finally, the high-resolution scanning electron microscopy (HRSEM) JEOL JSM-7800F was used to analyze the morphology of the composite templates and to obtain EDS maps (on MPS-HA sample).

## Results and Discussion

The chemical composition of hexagonal HA (within the ideal P6_3_/m space group) is Ca(I)_4_Ca(II)_6_(PO4)_6_(OH)_2_ [29]. The Ca(I) site is surrounded by six PO_4_^3−^ tetrahedral and coordinated by nine oxygen ions. The Ca(II) site is seven-coordinated with six oxygen ions from PO_4_^3−^ and one oxygen ion from OH^−^. In order to determine the effect of pore size on the growth and crystallization of HA nanostructures, we compare their deposition on/inside the two morphologically different nanostructured PS templates (n-, p-type) using a co-precipitation method. A schematic description of the mechanism that controls the morphology and size of HA nanostructures, due to the confinement of HAP precursor solution within the porous structure, is presented in Fig. 1b. On the basis of experimental observations, the growth processes on/within porous structures have been proposed (Fig. 1c(*i*)), i.e., the HA precipitation mechanism follows a series of events such as infiltration by capillarity (Fig. 1c(*ii*)), nucleation of the crystals on the rough porous template (Fig. 1c(*iii*)), followed by aggregation induced by capillary confined Ca^2+^ ions (Fig. 1c(*iv*)), and their corresponding growth (Fig. 1c(*v*)). The aggregation plays a highly relevant role in determining the final shape and size of HA particles [29]. As a consequence, the oriented attachment of HA grown with a regular flake-like shape (see Fig. 1c) can be observed. The above discussion reveals the significant role played by the nanostructured PS in the modification of particle size and morphology due to the confinement of the precursor solution.

### HA Powder Sample

**Fig. 2:**
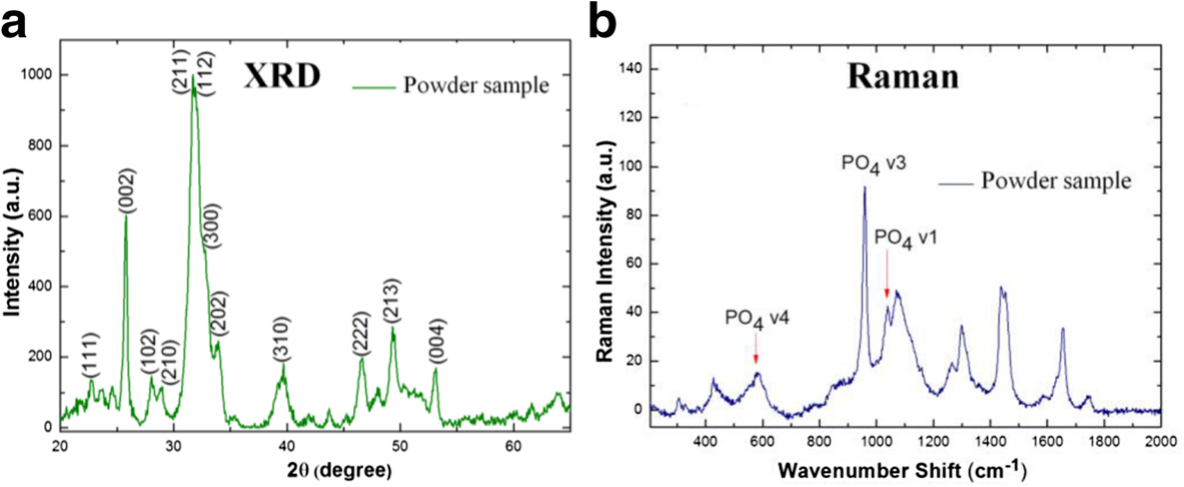
**a** XRD and **b** Raman spectra of HA powder sample

The HA powder obtained from co-precipitation during the synthesis (not attached to porous template) was analyzed by XRD and Raman spectroscopy, and its morphology/crystalline structure was determined by TEM. Figure 2a shows the XRD spectrum of the HA powder sample obtained with the method discussed above. Diffractogram shows well-defined HA peaks revealing its polycrystalline nature. The main diffraction peaks are in good agreement with the ICDD, PDF # 01072-1243 card and in conformity with the previously reported studies using different synthesis methods [40–42]. Indexed peaks correspond to the space group P6_3_/m of the hexagonal phase, consistent with the already reported work at around 100 °C [29]. Diffraction peaks in the present case are found to be relatively broader, which indicates smaller particle size.

Hydroxyapatite Raman vibrations are associated with the well-known internal four different tetragonal PO_4_^3−^ vibrational modes: *ν*_1_ correspond to a totally symmetric stretching mode of the tetrahedral PO_4_^3−^ group (P-O bond), *ν*_2_ is a doubly degenerate bending mode of the phosphate group (P-O-P bond), *ν*_3_ is a triply degenerate asymmetric stretching mode of the tetrahedral PO_4_^3−^ group (P-O bond), and *ν*_4_ is a triply degenerate bending mode of the PO_4_ group (O-P-O) [40, 41]. Figure 2b shows Raman spectra obtained from the HA powder sample. Three vibrational modes can be clearly identified. The analysis reveals a first band at 1045 cm^−1^ corresponding to the *ν*_3_ phosphate (PO_4_) mode. Another appears at 958 cm^−1^, corresponding to the *ν*_1_ phosphate (PO_4_) mode, and finally one more is revealed at 583 cm^−1^, which corresponds to the typical *ν*_4_ band (PO_4_) of hydroxyapatite [43–45].

**Fig. 3:**
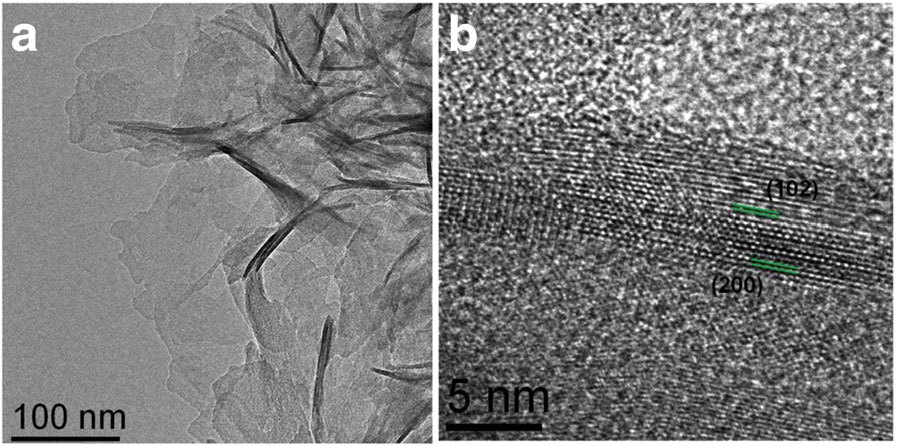
**a** TEM and **b** HRTEM images of HA obtained in powder

In order to identify the morphology and particle size of HA powder sample, Fig. 3a, b shows the low- and high-magnification TEM images, respectively. Figure 3b reveals the lattice fringes used to determine the inter-planar distance. Measured lattice spacing of 3.170 and 4.084 nm is found to be in agreement with the already reported spacing (corresponding to HA structure) of (102) and (200) planes, respectively. Crystal lattice parameters from the analysis of high-resolution images were found to be in agreement with those reported in the ICDD PDF # 01-072-1243 card. These results are consistent with the observed XRD analysis, shown in Fig. 2a.

### Mesoporous HA@PS-n and HA@PS-p Composite Samples

**Fig. 4:**
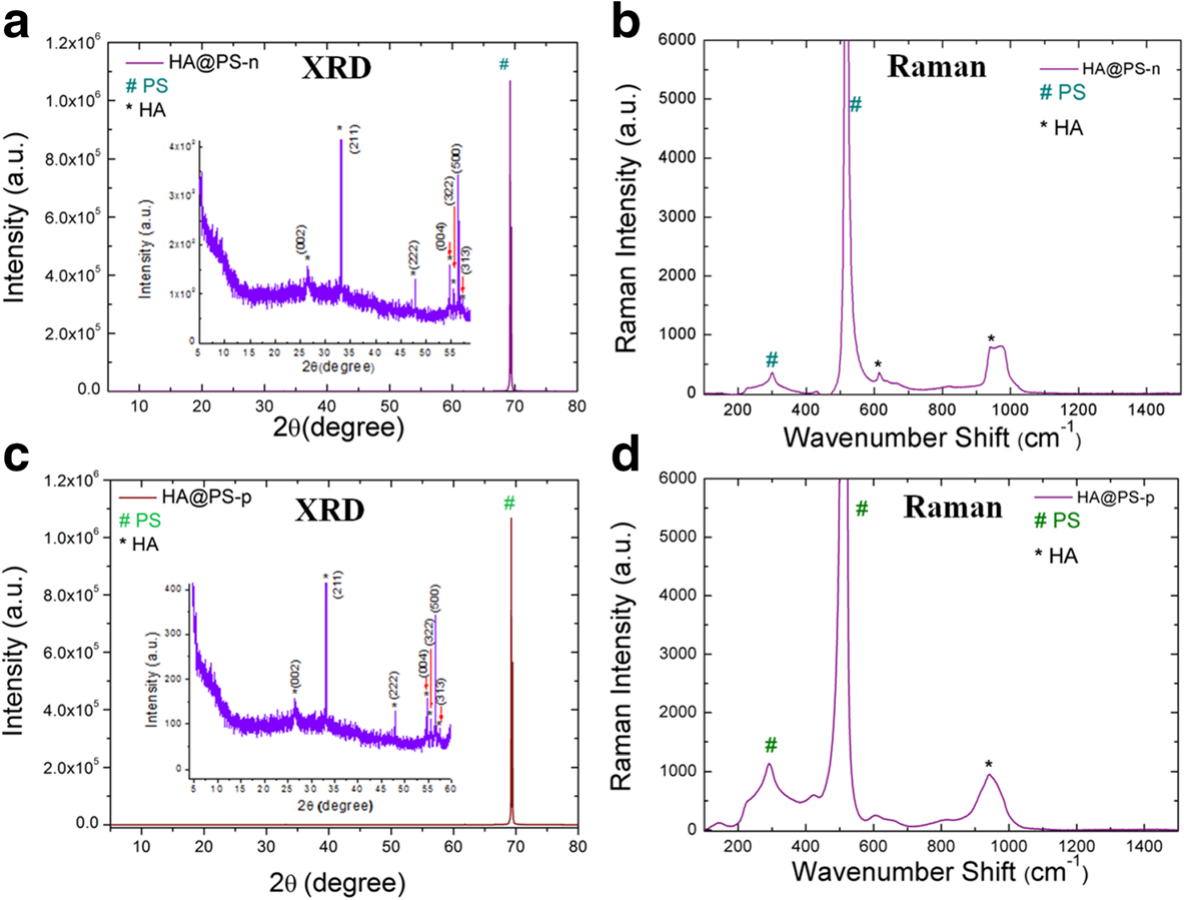
XRD and Raman spectrum of **a**, **b** HA@PS-n and **c**, **d** HA@PS-p composites

In the case of mesoporous silicon composites, two different PS substrates were used to induce the nucleation of HA crystals from co-precipitation method and to show the possible HA deposition/crystallization process at room temperature. In Fig. 4, the single XRD and micro-Raman study for the HA@PS-n (Fig. 4a, b) and HA@PS-p (Fig. 4c, d) samples is presented. In both cases, the observed diffraction peaks (see Fig. 4a, c) clearly show the formation of the HA nanoparticles, in agreement with the corresponding PDF # 01072-1243 card. According to these data, the main peaks, characteristic for the HA, were the isolated (002) planes, with a peak at 2*θ* = 26° and a broad peak, centered at 32.5°, which was an envelope of overlapping different (211), (112), (300), and (202) crystalline planes [21]. As the size of the individual crystal is less than 100 nm with many nanometric and randomly orientated crystals, X-ray signal shows broadening. Besides the HA crystalline phase, the sample reveals a characteristic silicon peak at 2*θ* = 69°.

Typical vibrational modes present in HA@PS-n and HA@PS-p samples are shown in Fig. 4b, d. Two groups of peaks at about 430 and 585–611 cm^−1^ were identified as *v*_2_ and *v*_4_ phosphate modes, characteristic of HA nanostructures. A peak at 961 cm^−1^, clearly detected in the samples HA@PS-n and HA@PS-p, was assigned to the *v*_1_ phosphate symmetric stretching of the HA, which is generally the strongest peak in the HA spectrum. The peaks measured at 520 and 303.6 cm^−1^ were also assigned to crystalline silicon [21]. Similar to XRD results, this analysis confirmed the room temperature crystallization of HA on porous silicon.

**Fig. 5:**
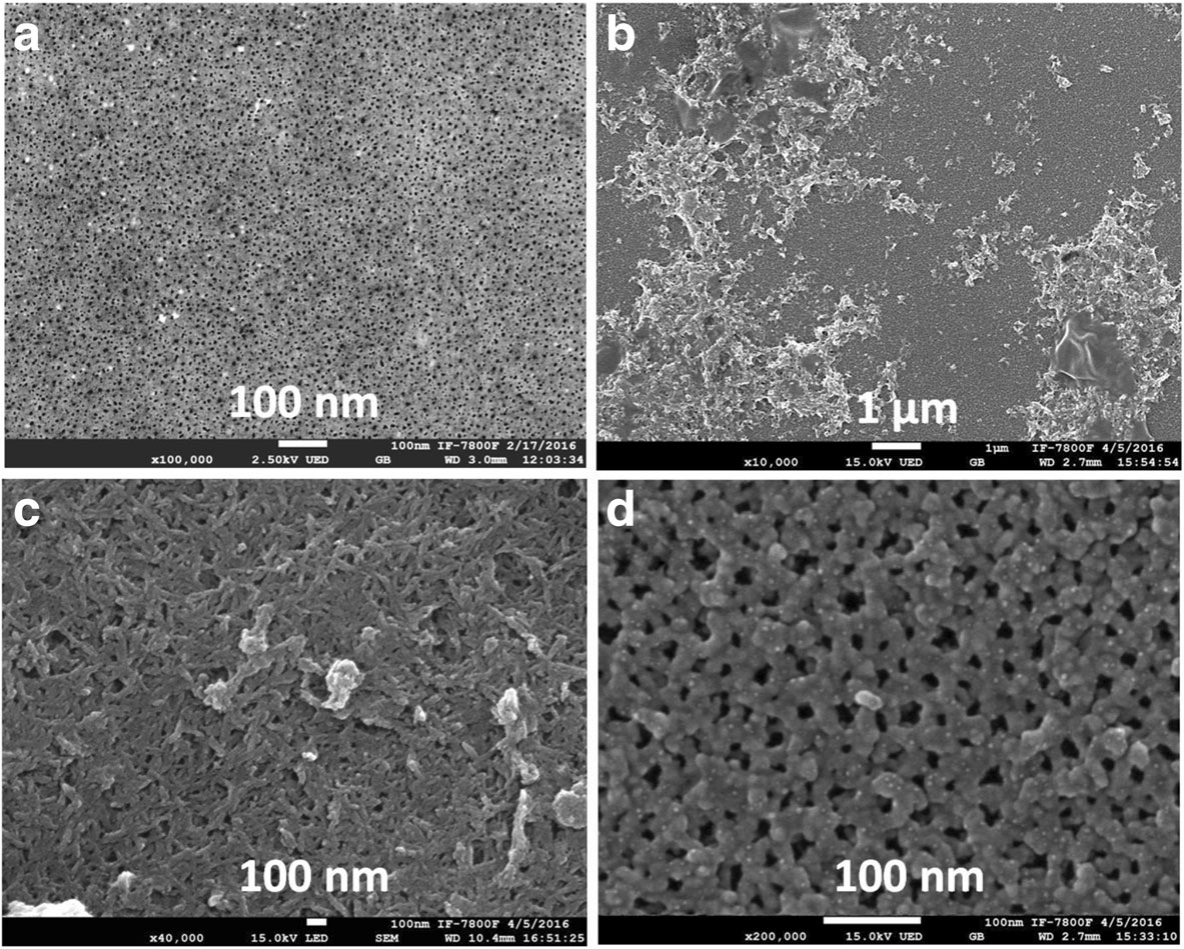
SEM images of HA@PS-n sample. **a** Bare porous silicon, **b**–**d** composite samples at different magnifications

The SEM images corresponding to HA@PS-n sample obtained by electrochemical etching are shown in Fig. 5. Figure 5a shows the top view of bare n-type porous silicon substrate. Figure 5b shows the HA nucleation and agglomeration in some parts of the porous template, formed by co-precipitation method, reaching a height of approximately 1 μm with respect to the PS surface. Due to small pore sizes (2 to 10 nm) of the PS surface, a complete infiltration within the porous silicon matrix was not obtained. Besides, the image of Fig. 5c shows that the HA particles are floccule-like, around 50 nm in diameter, and in some areas, the particles are well-agglomerated in clusters. Morphologically, the HA particle growth in/over PS is similar to others synthesized previously [20]. Due to increased porosity in the area where the sample is in contact with the precursor solution, the growth of hydroxyapatite is very different compared to the area where the solution is infiltrated by capillarity (see Fig. 5d). This indicates that the co-precipitation technique is viable to form a composite based on HA and PS at room temperature.

**Fig. 6:**
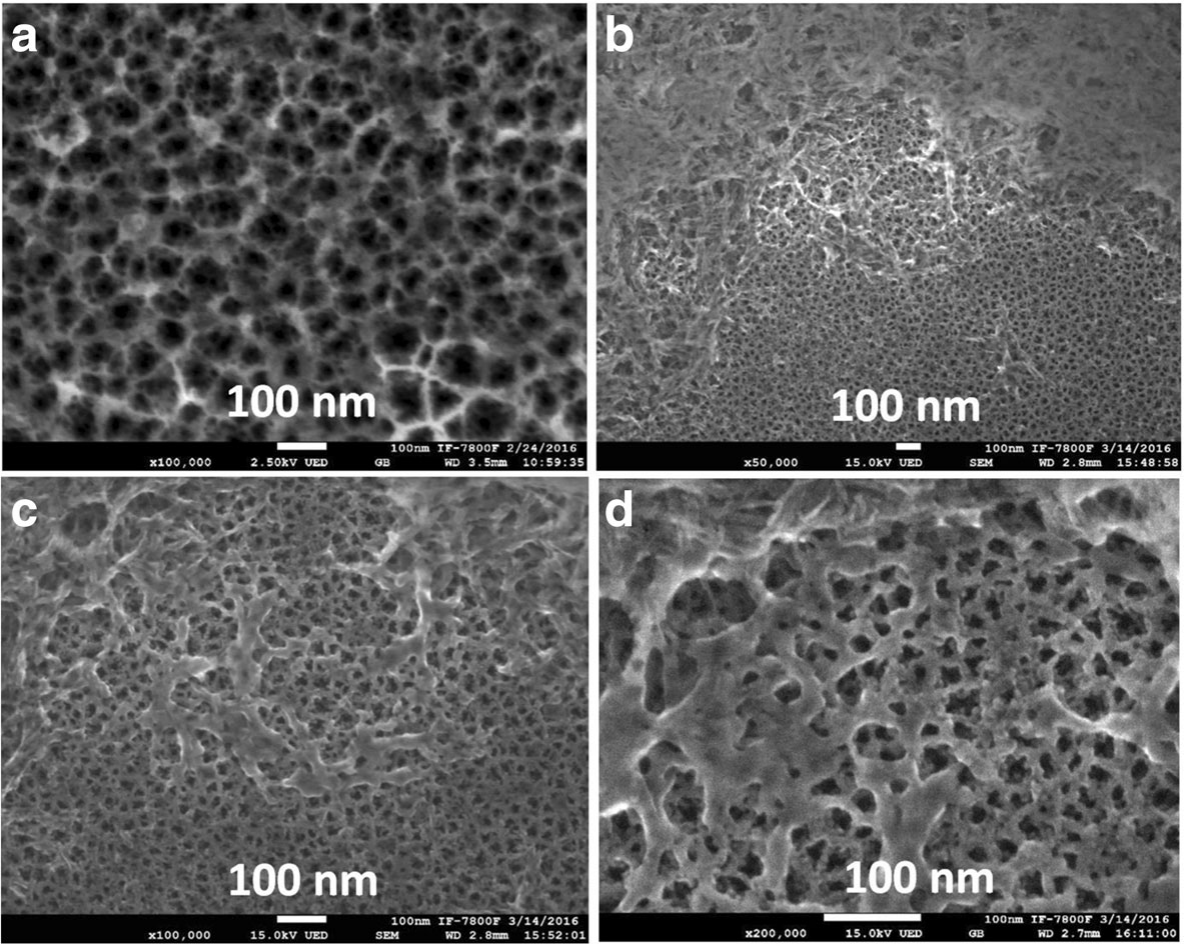
SEM images of HA@PS-p sample. **a** Bare porous silicon , **b**–**d** composite samples at different magnifications

Although HRSEM images of the HA@PS-p show similar morphology as compared to HA@PS-n sample, due to the difference in the porosity of the porous template, the nucleation into the pores is relatively more efficient than p-type porous structure. Figure 6a shows the surface view of bare p-type PS substrate. Figure 6b shows that the HA nucleation formed by co-precipitation method consists of agglomerates, in some parts of sample, reaching a height of approximately 2 μm with respect to the PS surface. Due to macropore sizes (50–100 nm) on PS surface, a partial infiltration within the porous silicon matrix was obtained. Besides, the image of Fig. 6c shows that the HA particles are interconnected fibers, around 100 nm in thickness, and in some areas into the porous template, the particles are well-agglomerated in small clusters (see Fig. 6d) of approximately 20 nm.

**Fig. 7:**
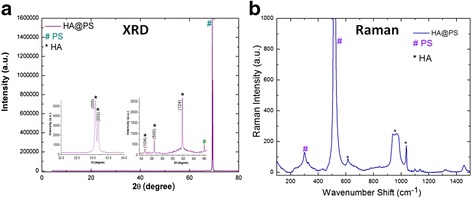
**a** XRD and **b** Raman spectra of HA@macroPS-n structure

Figure 7 shows the single XRD and micro-Raman (Fig. 7a, b) study for the composites formed with macroporous (sample denoted as MPS-HA) silicon substrates. Similar to the HA formed over the mesoporous substrate (HA@PS-n and HA@PS-p), diffraction and Raman [21, 39] peaks in the MPS-HA composite clearly show the presence of HA nanostructures.

**Fig. 8:**
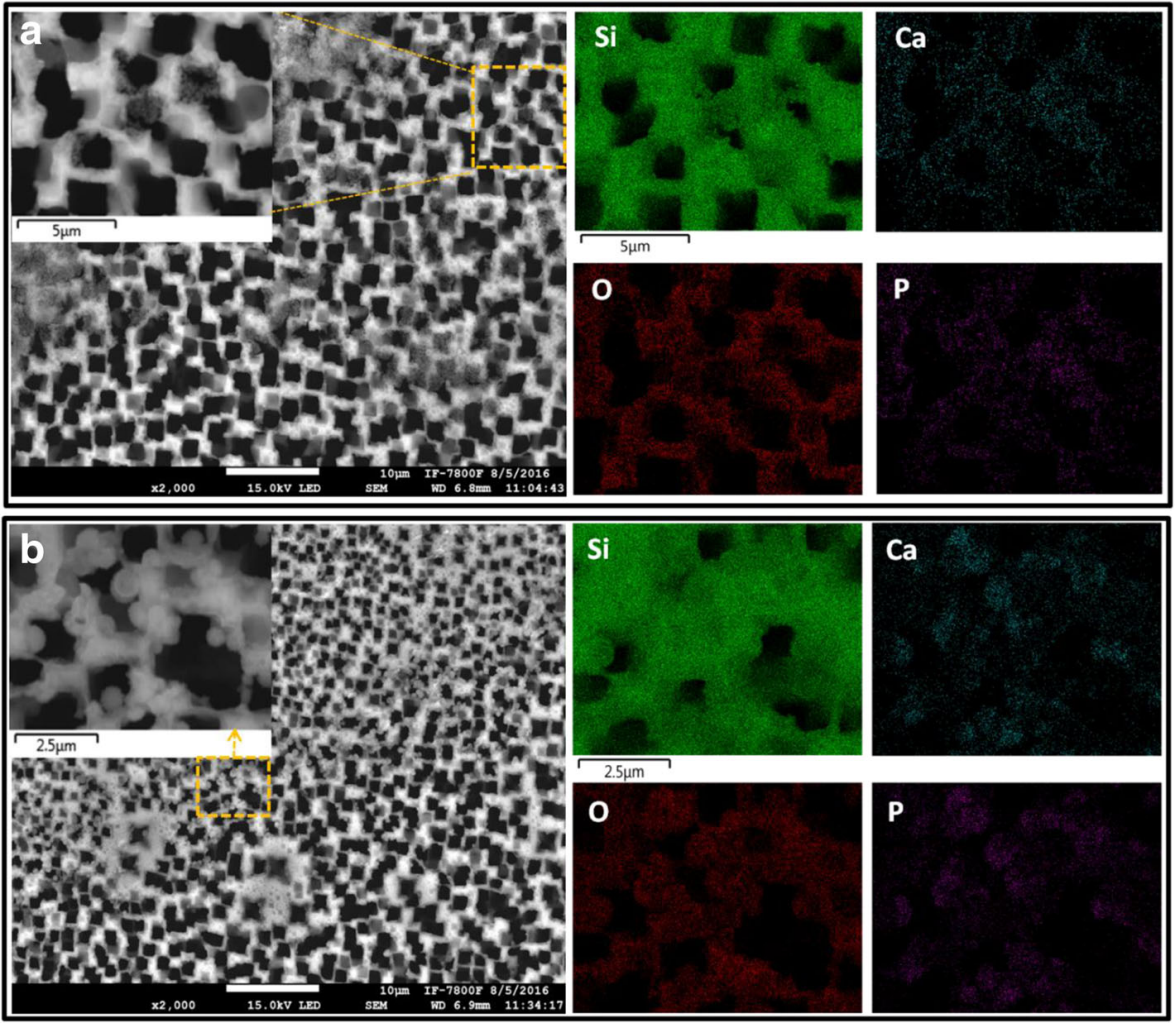
**a** High porosity zone of MPS-HA composite structure: (*left hand side*) top view taken from scanning electron microscopy (*inset* shows the magnified view); (*right hand side*) EDS mapping corresponding to the fibrous morphology confirms the nucleation and formation of HA within/over the MPS substrate. **b** Low porosity zone of MPS-HA composite structure: (*left hand side*) top view taken from scanning electron microscopy (*inset* shows the magnified view with the spherical morphology); (*right hand side*) EDS mapping corresponding to the spherical morphology confirms pore size and type the nucleation and formation of typical HA within/over the MPS substrate

In order to observe the HA nucleation in different pore dimensions in MPS structures, n-type graded macropores were fabricated employing an experimental setup which involves the simultaneous application of an electric and magnetic field along with an auxiliary metal electrode electrically connected to the negative contact of the lateral electric field (cathode) applied onto Si substrate [6, 38]. This configuration leads to the formation of linear gradient in pore size and thickness. Depending on the dimensions, the pore corners and walls of MPS structures reveal the formation of HA nanostructures of different sizes and structural shapes (Fig. 8a, b). In the present case, crystals formed within the pores are also confined due to pore size; however, they also grown from nanometer scale to typical spherical micrometric agglomerates [inset, Fig. 8b]. Different forms of agglomerations have been attributed to the different degree of surface roughness responsible for the nucleation of HA.

## Conclusions

Room temperature growth and crystallization of HA nanoparticles in/over partially oxidized porous silicon substrates have been demonstrated by co-precipitation method. Raman and XRD analyses of the composite samples revealed the characteristic phosphate peaks and the polycrystalline nature, respectively. Shape and pore size were favorable for the adhesion and growth of HA in porous structure, and the analysis confirms hydroxyapatite nanoparticles have a hexagonal structure. PS with different tunable optical and electrical properties can be used for the development of optical/electrical biosensors for monitoring HA growth. This opens the possibility to develop composite biomaterials for biomedical applications at room temperature or physiological temperature based on hydroxyapatite nanocompounds.
